# Effectiveness, feasibility, and acceptance of a general practice-based supportive intervention for dementia caregivers: the AD HOC trial

**DOI:** 10.1007/s41999-026-01437-7

**Published:** 2026-02-25

**Authors:** Gino Bopp, Thomas Grischott, Giuseppe Pichierri, Stephanie Greindl, Stefan Gysin, Oliver Senn, Thomas Rosemann, Stefan Neuner-Jehle

**Affiliations:** 1 Institute of Primary Care, University Hospital Zurich, University of Zurich, Sonneggstrasse 6, 8091 Zurich, Switzerland; 2 Faculty of Health Sciences and Medicine, University of Lucerne, Frohburgstrasse 3, 6002 Lucerne, Switzerland

**Keywords:** Dementia, Informal caregiver, Caregiver burden, Zarit Burden Interview, Quality of life, Non-physician staff

## Abstract

**Purpose:**

This study evaluated the effectiveness, feasibility, and acceptance of a caregiver-need-driven intervention to reduce caregiver burden among informal caregivers of people with dementia, delivered by non-physician medical staff of general practices in Switzerland.

**Methods:**

In this prospective, single-group, pre-post superiority trial, non-physician medical practice staff were trained to deliver a supportive intervention for informal dementia caregivers involving three home visits and optional home-based and/or telephone counseling. Comparator was the "usual care" arm of the randomized DE-REACH trial in a similar setting. Primary outcome was increase in caregiver burden at six months, measured by the Zarit Burden Interview (ZBI) and analyzed by one-sample superiority *t*-test. Secondary outcomes included changes in quality of life. Hierarchical regression served to identify associations between outcomes and participant characteristics.

**Results:**

Among 32 non-physician medical practice staff (96.9% female, mean 14.6 years of professional experience) and 55 informal caregivers (69.1% female, mean age 70.0 years), the intervention proved superior to usual care by significantly attenuating the increase in caregiver burden (mean ZBI score increase 2.65 [95% confidence interval: -∞ to 4.10] vs. 7, *p* = 0.017). There was no significant change in quality of life during the intervention, which was well feasible and accepted by caregivers and health professionals.

**Conclusion:**

This study demonstrated that a scalable, caregiver-need-driven intervention by trained non-physician medical staff in general practices mitigated the rise in caregiver burden over six months. The intervention holds potential to enhance the efficacy of general practice teams in supporting the frequently overlooked dementia patient-caregiver dyad, thereby improving their well-being and the quality of care provided.

**Trial Registration:**

ISRCTN55348828, registered 12/10/2021.

**Supplementary Information:**

The online version contains supplementary material available at 10.1007/s41999-026-01437-7.

## Key Summary Points

**Aim:**

This study evaluated the effectiveness, feasibility, and acceptance of a caregiver-need-driven intervention to reduce caregiver burden among informal caregivers of people with dementia, delivered by non-physician medical staff of general practices in Switzerland.

**Findings:**

The intervention proved superior to usual care by significantly attenuating the increase in caregiver burden, measured by the Zarit Burden Interview (ZBI) (mean ZBI score increase 2.65 [95% confidence interval: − ∞ to 4.10], compared to an increase of 7 under usual care in similar settings). The interventions was well feasible and accepted by both caregivers and health professionals.

**Message:**

The scalable, caregiver-need-driven, home-based intervention by trained non-physician medical staff in general practices mitigated the rise in dementia caregiver burden over six months and has the potential to improve caregiver well-being and the quality of care for people with dementia.

**Supplementary Information:**

The online version contains supplementary material available at 10.1007/s41999-026-01437-7.

## Background

Dementia is a frequent disorder among the aging population, with a worldwide prevalence of 5–7% in those aged 60 years or older [1]. While an estimated 150,000 people were affected in Switzerland alone in 2017, this number is expected to double until 2050 due to population growth [2]. Alzheimer's disease, with 60–70% the most common form of dementia, usually progresses from mild and largely unapparent cognitive impairment to severe and disabling limitations, including loss of judgement, orientation difficulties, changes in personality and behavior, and problems with language and communication [3, 4]. As the severity of dementia increases, patients need more and more support and care, often 24 h a day and not infrequently for many years [2]. This care effort is typically provided by the patients’ relatives—mostly females, in two out of three cases spouses—and can translate into a considerable physical, mental and social burden that affects the caregivers’ own quality of life [5, 6]. From a preventive health perspective, it is therefore essential not only to focus on people with dementia, but also to address the challenges faced by their informal caregivers and to provide them with appropriate support.

In research on the care burden of informal caregiving [7–9], the term *caregiver burden* is not well defined, but it usually includes the notion of a self-perceived persistent imbalance between multiple strains and available resources, and is heavily influenced by the patient’s degree of dependency, functional limitations, and behavioral symptoms of dementia [10–12]. Despite some positive effects of caregiving, such as "spiritual” or "personal growth" and "feelings of mastery", many caregivers suffer from negative effects such as anxiety, insecurity, overwork, feelings of helplessness and feelings of being overwhelmed [13–15]. Informal caregivers of dementia patients, often called the “invisible second patient”, experience higher rates and severity of depression and anxiety compared to non-caregivers, with women being particularly affected [16–19].

Various existing interventions aim at reducing the informal caregivers’ burden of care [20–22]. Individualized, problem-oriented approaches were shown to be particularly effective in reducing caregiver burden [23, 24]. For instance, the multi-component REACH II intervention—grounded in Schulz’s theoretical framework model for the stress–health process in informal dementia caregivers [25]—improved caregivers’ self-perceived health by providing need-driven, individualized support through information provision, skills training, stress management techniques, problem-solving strategies, role-playing, and telephone support [26]. Its German adaptation DE-REACH proved effective to stabilize burden of care over time and led to a better quality of life for the caregivers [27].

In our study, we explored whether a stabilizing effect on caregiver burden as found in REACH II and DE-REACH can be achieved with fewer resources in terms of staff and time. In order to ensure comparability, we used the same instrument to measure caregiver burden, but as a novel aspect of our study, the intervention was delivered by non-physician medical practice staff (npMPS) from general practices, who are ideally suited to provide and coordinate such individualized support due to their familiarity with the problems and needs of those affected. We hypothesized that our supportive intervention would (i) result in a smaller increase in caregiver burden than the natural progression, while (ii) stabilizing the quality of life (QoL) of informal caregivers of people with dementia during the intervention period, and (iii) prove feasible and acceptable for practical implementation.

## Methods

### Study design

**Fig. 1:**
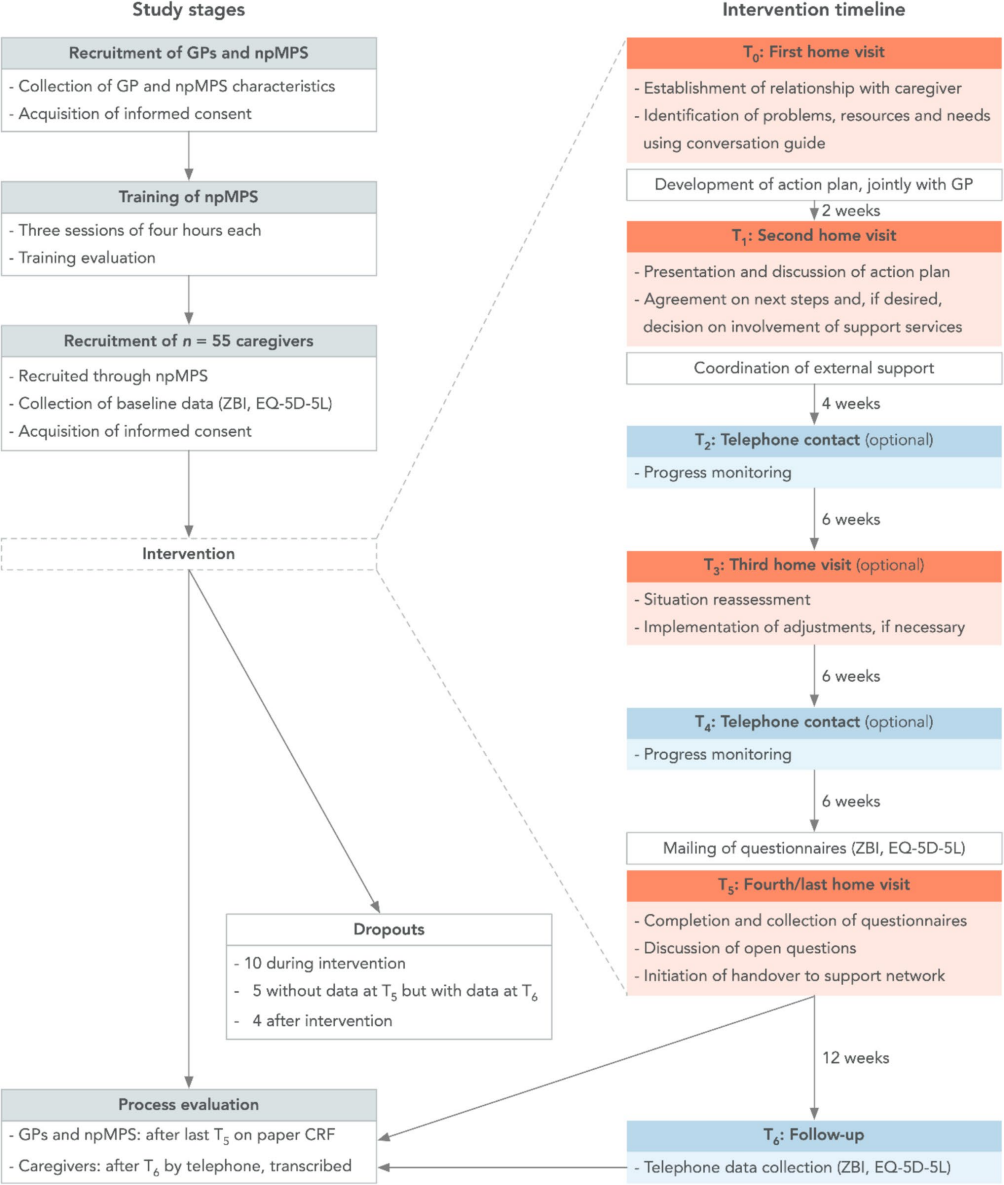
Study flowchart. Left: Sequence of study stages. Right: Intervention steps. Intervals between *T*_0_ (baseline) and *T*_6_ (end of follow-up) are guide values. *GP* general practitioner; *npMPS* non-physician medical practice staff; *ZBI* Zarit Burden Interview; *EQ-5D-5L* European Quality of Life 5 Dimensions 3 Levels questionnaire; *CRF* case report form

This pragmatic study in outpatient general practice was designed as a prospective, single-group, pre-post superiority trial with “usual care” as in the control arm of the DE-REACH trial [27] as comparator. The study design and materials were developed by study team members with expertise in both primary care practice and research, in collaboration with an advisory board of experts in dementia or chronic care (see Acknowledgements). Figure 1 (left panel) shows the study flowchart.

Ethics approval was not required for this study because it does not fall within the scope of the Human Research Act, as confirmed by the Ethics Committee Zurich (BASEC No. Req-2021-00280). The study was registered on 12/10/2021 as ISRCTN55348828, and reporting follows the CRISP consensus statement for studies in primary care [28].

### Participants

In Switzerland, healthcare roles follow distinct educational pathways and responsibilities. Medical practice assistants (MPAs) complete a 3-year vocational apprenticeship and perform core clinical and administrative tasks, akin to medical assistants in the USA. Medical practice coordinators (MPCs) represent an advanced role and require at least 3 years of professional experience and successful completion of a federal examination following modular training in clinical care (e.g., chronic disease management) or practice management (e.g., leadership, quality improvement). Their scope includes coordination and leadership functions, exceeding that of MPAs or typical US medical assistants. Advanced practice nures (APNs) are registered experts with a Master of Science in Nursing and demonstrated supervised clinical practice, with qualifications and scope aligned to US nurse practitioners.

Participants in our study were: (i) npMPS from general practices in German-speaking Switzerland who had completed training as MPA, MPC, or APN, and who were expected to remain employed in their practice for at least 6 months; and (ii) adult informal caregivers of patients diagnosed with dementia (see below), who were recruited by the participating npMPS. All participants provided informed consent.

### Recruitment of general practices

Between September 2021 and October 2022, we contacted 2116 general practitioners (GPs) at congresses, through physician networks, and via postal mail, inviting them to participate with their npMPS in our study. Each participating practice was offered a base compensation of CHF 2000 (approximately USD 2100). Participating GPs and npMPS provided written informed consent.

### Training of npMPS

The npMPS were trained by the study team and experts working in dementia care and counseling (Fig. 1 (left panel), Supplementary Table 1). The 12-h training was divided into three sessions. In the first two sessions, prior to their interactions with the informal caregivers, the npMPS received basic information about dementia and were instructed about their study tasks, in particular how to use a conversation guide based on existing tools [27, 29] to learn about the caregivers' situations, and how to develop individual action plans. The npMPS also prepared lists of regional support and relief services and were trained to reach out to our experts for assistance when necessary. In the third training session, the npMPS each reported on one of the recruited caregivers and shared their experiences under the supervision of the dementia experts. Training success was assessed using Kirkpatrick’s 4-level training evaluation model [30] (Supplementary Table 2).

### Eligibility and recruitment of informal caregivers

Caregivers were recruited by the npMPS from September 2021 to February 2023. Inclusion criteria were: (i) aged ≥ 18 years, and (ii.a) perceived by the npMPS or their GP to be the primary informal caregiver of a person diagnosed with Alzheimer's disease, dementia or developing dementia, or (ii.b) named by such a person as their primary informal caregiver. Caregivers were excluded if (i) deemed by the npMPS to be unable to answer study-specific questions, complete questionnaires, or provide informed consent due to cognitive impairment or language barriers, or (ii) if they had a life expectancy of < 3 months. Written informed consent, as well as baseline outcome measures, were obtained from all included caregivers before their first study activities.

### Study intervention

The interventions of approximately 6 months for the individual caregiver took place between January 2022 and December 2023. Follow-up continued individually for an additional 3 months and was completed for all informal caregivers by March 2024.

The different steps of the intervention are shown in Fig. 1 (right panel). At the first visit (*T*_0_), the npMPS used the conversation guide to familiarize themselves with the caregivers’ specific situations, resources, challenges, and needs (Supplementary Table 3). In consultation with their supervising GPs, the npMPS then developed an individualized action plan, which was discussed with the informal caregivers two weeks later during the second home visit (*T*_1_). If desired by the caregivers, the npMPS then involved and coordinated regional support services. Further home visits (*T*_3_) and telephone calls (*T*_2_, *T*_4_) followed at regular intervals to monitor progress and adjust support services as needed. The active intervention ended after 6 months with the fourth home visit (*T*_5_).

### Outcome measures

The *primary outcome* was the increase in caregiver burden within the 6 months between the first and last home visit, measured using a validated German version of the Zarit Burden Interview (ZBI) [31], a frequently applied measurement instrument for the subjective burden of caregivers of dementia patients [32]. In the ZBI, caregivers rate 22 statements about the care recipient's behavior, their own mental and physical health, financial situation, work intensity, relationship with the care recipient, and support from family members. Higher scores indicate a greater burden. Total scores > 24 have been suggested as high and scores ≤ 24 as low burden [33].

*Secondary outcomes* were the increase in the ZBI score within 9 months and the changes in the caregivers’ quality of life within 6 and 9 months from the first home visit, assessed using a validated translation of the EQ-5D-5L questionnaire [34]. This tool captures health-related quality of life in five dimensions (5D) with five possible levels (5L) and also requires respondents to rate their current health status on a visual analogue scale (VAS) from 0 (= worst) to 100 (= best possible health status).

The following *implementation outcomes* were collected using customized, semi-structured questionnaires with fields for free-text answers; from GPs and npMPS after completion of all their interventions (i.e., after their last *T*_5_) directly on paper, and from the informal caregivers at the end of their individual follow-up (i.e., at T_6_) by phone and then transcribed on paper: Acceptance of the intervention model, time required, feasibility of, and barriers to the intervention. We also recorded the participation rate among all caregivers contacted as well as the dropout rate among all caregivers who initially participated in the study.

### Data collection, data management and handling of dropouts

All data collected on paper case report forms were transferred into the secure web application REDCap (Research Electronic Data Capture [35]) using double data entry. Data from caregivers who dropped out of the study were analyzed up to the time of dropout. Dropouts were recorded along with reasons, whenever possible, to assess the potential impact of the intervention on the dropout rate.

### Sample size

The mean natural increase of the ZBI score within 6 months was reported to be roughly ${\mu }_{0}= 7$ in a comparable population [27]. We postulated that our intervention would reduce this increase by at least $\delta =2$, which we considered the minimal clinically important difference (MCID). Therefore, we tested H_0_: ∆ ZBI ≥ ${\mu }_{0}$ – $\delta$ = 7 – 2 = 5 vs. H_A_:∆ ZBI < 5 with a one-sided one-sample *t*-test. To achieve a power of $1-\beta =80\%$ for the specific alternative ∆ ZBI = 0 and with a significance level of $\alpha =5\%$, and further assuming a standard deviation $s = 10$ as in the validation study [31], an individual pre-post correlation $r=0.7$, an intra-cluster correlation $\rho = 0.04$, and clusters of size ${n}_{c} = 5$ (5 caregivers per npMPS), the required sample size was 18 caregivers. With an estimated maximal dropout rate of $d = 40\%$, this amounted to 30 caregivers in 6 clusters.

### Data analysis

Descriptive characteristics of npMPS and caregivers were presented as numbers and proportions or means with standard deviations as appropriate (Tables 1, 2).

**Table 1:** Non-physician medical practice staff (npMPS) characteristics

Characteristic	All *n* = 32	Active *n* = 21	*p*-Value^a^
Sex, *n* (%)			0.344
Female	31 (96.9)	21 (100.0)	
Age, years, mean (SD)	38.8 (11.8)	40.9 (12.1)	0.175
Education,* n* (%)			0.035
Medical practice assistant (MPA)	10 (31.2)	4 (19.0)	
Medical practice coordinator (MPC)	12 (37.5)	10 (47.6)	
Advanced practice nurse (APN)	5 (15.6)	5 (23.8)	
Any of the above, with additional qualification^b^	5 (15.6)	2 (9.5)	
Professional experience, years, mean (SD)	14.6 (11.0)	15.8 (11.3)	0.396
Type of practice, *n* (%)			0.555
Group practice	24 (75.0)	16 (76.2)	
Two-physician-practice	6 (18.8)	3 (14.3)	
Single practice	2 (6.2)	2 (9.5)	

*SD* standard deviation

^a^Comparison of active npMPS with dropouts (= 11 npMPS who did not recruit caregivers)

^b^Additional qualifications included nurse (*n* = 3), social worker (*n* = 1), and nutritionist (*n* = 1)

There were no missing data

**Table 2:** Informal caregiver characteristics and outcome baselines

Characteristic	*T*_0_ *n* = 55	*T*_5_ *n* = 40	*T*_6_ *n* = 41	*p*-Value^a^
Sex, *n* (%)				0.754
Female	38 (69.1)	27 (67.5)	26 (63.4)	
Na	0	0	0	
Age, years, mean (SD)	70.0 (13.2)	69.7 (12.2)	70.4 (10.8)	0.768
Na	3 (5.5)	0	3 (7.3)	
Education, *n* (%)				0.694
Compulsory school	16 (29.1)	13 (32.5)	12 (29.3)	
High school or vocational training	26 (47.3)	17 (42.5)	20 (48.8)	
University	12 (21.8)	9 (22.5)	8 (19.5)	
Na	1 (1.8)	1 (2.5)	1 (2.4)	
Civil status, *n* (%)				0.878
Single	2 (3.6)	1 (2.5)	1 (2.4)	
Married	48 (87.3)	35 (87.5)	36 (87.8)	
Registered partnership	1 (1.8)	1 (2.5)	1 (2.4)	
Divorced	3 (5.5)	2 (5.0)	2 (4.9)	
Na	1 (1.8)	1 (2.5)	0	
Nationality, *n* (%)				0.732
Swiss	51 (92.7)	37 (92.5)	38 (92.7)	
Other	2 (3.6)	2 (5.0)	1 (2.4)	
Na	2 (3.6)	1 (2.5)	2 (4.9)	
Relation to care receiver, *n* (%)				0.680
Spouse/partner	37 (67.3)	29 (72.5)	30 (73.2)	
Child	10 (18.2)	8 (20.0)	9 (22.0)	
Other relative	2 (3.6)	2 (5.0)	1 (2.4)	
Na	6 (10.9)	1 (2.5)	1 (2.4)	
Living situation, *n* (%)				1.000
With care receiver	47 (85.4)	34 (85.0)	35 (85.4)	
With other person(s)	6 (10.9)	4 (10.0)	4 (9.8)	
Alone	1 (1.8)	1 (2.5)	1 (2.4)	
Na	1 (1.8)	1 (2.5)	1 (2.4)	
ZBI baseline score, mean (SD)	27.1 (11.4)	25.1 (12.0)	25.0 (11.8)	0.027
Na	0	0	0	
QoL WIS baseline, mean (SD)	0.927 (0.097)	0.925 (0.108)	0.923 (0.109)	0.768
Na	3 (5.5)	2 (5.0)	2 (4.9)	
QoL VAS baseline, mean (SD)	77.9 (14.4)	77.3 (13.3)	77.7 (12.8)	0.614
Na	2 (3.6)	2 (5.0)	2 (4.9)	

*Na* missing; *SD* standard deviation; *ZBI* Zarit Burden Interview; *QoL* quality of life; *WIS* weighted index score (of the EQ-5D-5L instrument); *VAS*: visual analogue scale (of the EQ-5D-5L instrument)

^a^Comparison of responders at *T*_5_ with others (= 15 informal caregivers without data at *T*_5_)

The primary outcome was presented as mean with standard deviation (SD) and with a one-sided 95% confidence interval (CI), and compared using a one-sample superiority *t*-test against the ZBI score progression under usual care as reported by Berwig et al. [27]. The analysis was adjusted for missing values and clustering by npMPS (see below). The increase in the ZBI score over 9 months was analyzed similarly, this time assuming a mean natural increase of ${\mu }_{0}= 8$ and again a MCID of $\delta =2$. For the changes in quality of life within 6 and 9 months, we calculated two-sided 95% CIs using the French TTO value set to compute the weighted index scores (WIS) of the EQ-5D-5L instrument. To explore possible associations between ZBI and EQ-5D-5L outcomes on the one hand and characteristics of the npMPS and caregivers on the other, we used hierarchical regression models with random intercepts to account for clustering by npMPS. The analysis was intention-to-treat, with missing values multiply imputed across *m* = 30 datasets. The impact of imputation was assessed by “pragmatic” complete case sensitivity analysis, in which we allowed up to 3 missing items (out of 22) per time point in the ZBI and calculated the ZBI sum scores excluding the missing items.

Implementation outcomes (Likert scales, time required, response rates) were evaluated using descriptive statistics.

Statistical analyses were carried out with the R statistical package, version 4.4.0 [36], using the packages eq5d 9.15.3, tableone 0.13.2, mice 3.16.0, miceadds 3.17-44, and lme4 1.1-35.3. MAXQDA version 24.3 [37] was used for qualitative analyses.

## Results

### Study population (npMPS and informal caregivers)

Characteristics of the 32 npMPS (including 11 dropouts before *T*_0_) and the 55 informal caregivers (including 15 without data at *T*_5_ and 14 without data at *T*_6_) are shown in Tables 1 and 2.

The 11 npMPS who did not recruit caregivers and were therefore classified as dropouts differed from the actively recruiting npMPS only in a slightly different distribution of their education. Reasons given for dropping out of the study were: high workload due to staff shortage; change of job; too few informal caregivers known in their practice database who would have been eligible for the study.

The 15 informal caregivers who did not provide data at *T*_5_ had higher baseline ZBI scores, but otherwise did not differ from all other participating caregivers. Of the latter, a further 4 dropped out by the end of the follow-up at *T*_6_; conversely, 5 caregivers without data at *T*_5_ still provided data at *T*_6_. In most cases, dropouts were due to the care receiver’s admission to a care facility (9/15, 60.0%), in individual cases due to death, relocation, or long-term hospitalization of the care recipient, or to hospitalization or excessive strain on the caregiver. In one case, no reason was given.

The burdensome challenges most frequently self-reported by informal caregivers were the impact on their own health (reported by 29 out of 46, or 63.0% of caregivers), dealing with agitation, resistance, or other difficulties in social interaction with the person with dementia (29/46, 63.0%), and the lack of free time and opportunities to retreat and recharge (23/45, 51.1%).

### Primary and secondary outcomes

**Fig. 2:**
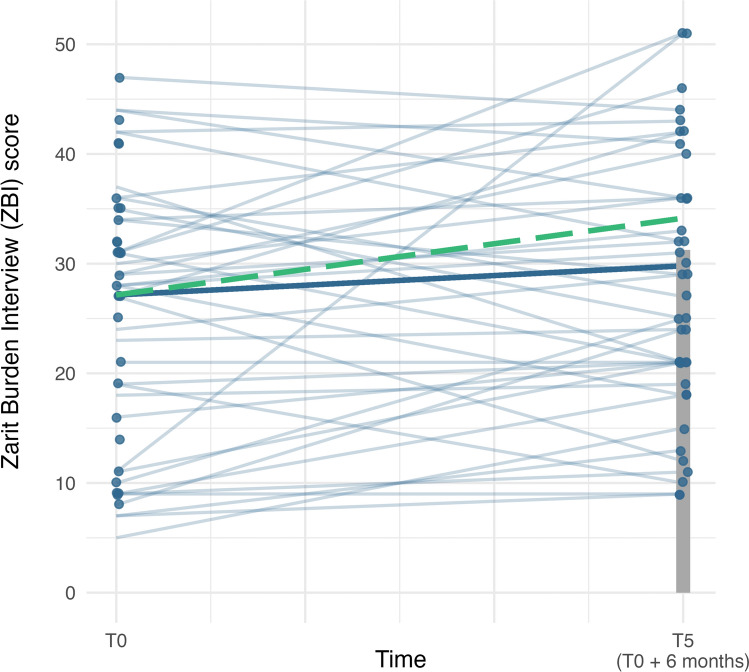
ZBI trajectories up to the time *T*_5_ of the primary outcome. Thin lines connect ZBI scores of the same caregiver, the thick blue line shows the mean ZBI increase, the dashed green line shows the natural ZBI increase under usual care, and the grey vertical line represents the one-sided 95% confidence interval for the ZBI score at *T*_5_

Figure 2 shows the ZBI scores of each caregiver at *T*_0_ (baseline) and at *T*_5_ (last home visit 6 months after *T*_0_). The ZBI scores increased by an average of 2.65 (thick blue line), with a one-sided 95% CI upper limit of 5.49 in the uncorrected complete case analysis. After multiple imputation of missing values and accounting for the clustered data structure, the adjusted CI had an upper limit of 4.10, which lies 2.90 points below the expected increase of 7 under usual care (dashed green line), corresponding to a *p*-value of 0.017 of the (adjusted) superiority *t*-test with a superiority margin of 2 points.

(Ancillary note: There was one notable outlier whose ZBI score increased by 40 from an initial value of 11 to a score of 51 after 6 months. Without this outlier, the upper limit of the adjusted CI would have been 3.00, corresponding to a *p*-value of 0.003 of the adjusted superiority *t*-test.)

Table 3 summarizes the primary and all secondary effectiveness outcomes, including quality of life.

**Table 3:** Effectiveness results

Domain	Variable	Outcome	*n*	Mean (SD)	Adjusted CI^a^	*p-*Value^b^
Burden	ZBI	Increase *T*_0_ → *T*_5_	40	2.65 (10.66)	]-∞, 4.10]	0.017
		Increase *T*_0_ → *T*_6_	41	0.54 (10.08)	]-∞, 2.41]	< 0.001
Quality of life	WIS	Change *T*_0_ → *T*_5_	34	– 0.014 (0.088)	[ – 0.064, 0.001]	0.057
		Change *T*_0_ → *T*_6_	39	– 0.020 (0.126)	[ – 0.067, 0.011]	0.150
	VAS	Change *T*_0_ → *T*_5_	37	– 1.57 (15.28)	[ – 8.16, 3.67]	0.446
		Change *T*_0_ → *T*_6_	39	0.72 (14.23)	[ – 4.88, 5.06]	0.971

*ZBI* Zarit Burden Interview; *WIS* weighted index score (of the EQ-5D-5L instrument); *VAS* visual analogue scale (of the EQ-5D-5L instrument)

^a^95% confidence intervals after multiple imputation of all data for *n* = 55 informal caregivers and accounting for clustering by non-physician medical practice staff

^b^*p*-values of adjusted superiority *t*-tests (with minimal clinically important difference $\delta =2$) of ${\mu }_{0}= 7$ (*T*_5_) and ${\mu }_{0}= 8$ (*T*_6_), respectively, for burden outcomes, and of two-sided equality *t*-tests in case of the quality of life outcomes

In the six multivariable hierarchical regression models to explore associations between the ZBI score increases or the WIS and VAS value changes within 6 and 9 months on the one hand and the characteristics of the caregivers and the npMPS listed in Tables 1 and 2 on the other hand, no significant associations were found (Supplementary Table 4). The smallest *p*-value was 0.121 for a potential association between the ZBI score increase after 9 months and the living situation of the caregiver.

### Implementation outcomes

#### Recruitment

Recruitment of general practices and GPs proved to be difficult and required the recruitment phase to be extended by 6 months. Reasons given by GPs for not participating included concerns about the additional resources needed for study activities on top of the extra effort required by the ongoing COVID-19 pandemic, and the lack of specialist staff in general practice. Of all GPs contacted, 1.5% agreed to participate in the study, usually with one, and in exceptional cases two, volunteer npMPS from their practice. Of all informal caregivers contacted, 60.6% (37 out of 61) chose to enroll in the trial, based on data from the two-thirds of the participating practices that provided this information.

#### Time requirements for npMPS

Per informal caregiver, the npMPS estimated an average of 243 min (SD = 101.2, range = 92–400, *n* = 16 responders) spent on direct counseling and 76 min (34.6, 20–120, 19) spent on services in the absence of the caregiver (planning, coordination of activities, consulting with their GPs).

#### Feasibility and acceptance by GPs and npMPS

Most of the participating GPs felt that our supportive counseling approach was practical (16/17, 94.1%) and added value to their practice (15/16, 93.8%), and they planned to establish the new npMPS role in their practice on a permanent basis (12/17, 70.6%) (Supplementary Table 5). The main barriers to the intervention, according to the GPs’ free-text feedback, were the non-reimbursable costs in the Swiss tariff system, the lack of staff, and limited time resources. The majority of the responding npMPS (14/20, 70.0%) felt that their new role had created a benefit for the informal caregivers to whom they had provided counseling. More than half of them (12/19, 63.2%) saw added value in their new counseling responsibilities for themselves, and even more (13/18, 72.2%) wished to continue this type of counseling after the trial. The individual elements of counseling (time allowance, accessibility of the GP, sequence of intervention steps, training and materials) received approval rates in the 70–90% range, and almost all (18/20, 90.0%) of the responding npMPS found the program feasible overall (Supplementary Table 6).

#### Acceptance of intervention by informal caregivers

Of the *n* = 42 informal caregivers who expressed an opinion, 40 considered the npMPS to be competent (95.2%), 32 out of 40 found the counseling to be helpful (80.0%), and 38 of 42 would recommend the project to others (90.5%). Most informal caregivers (35/41, 85.4%) considered it important that the GPs were involved in the planning of the counseling sessions, while considerably fewer considered home visits to be important (22/38, 57.9%) (Supplementary Table 7).

## Discussion

### Principal findings

Our general practice-based intervention for informal caregivers of dementia patients proved superior to usual care for this neglected and burdened population. The intervention led to a significantly smaller increase in informal caregiver burden compared to the reference population in the control arm of the DE-REACH trial, the only other German-language trial of comparable design [27]. In contrast to this reference population, where health-related quality of life declined, we found no reliable evidence of a decline in the quality of life of the informal caregivers throughout the intervention and follow-up, which could indicate that the intervention not only mitigated the increase in caregiver burden but also may have had a stabilizing effect on caregivers’ quality of life. Our intervention was well accepted and considered feasible by npMPS and the informal caregivers.

### Comparison with the literature

Our sample of informal caregivers was slightly younger (mean age 70.0 vs. 73.4 years) but otherwise similar to the control arm of the DE-REACH trial. The baseline burden of our study population (27.15 out of a possible 88 points on the ZBI scale) was almost identical to that of the DE-REACH controls (28.16 points), which we interpret as evidence that potential additional stressors from the COVID-19 pandemic had subsided sufficiently by the start of our intervention so as not to compromise the comparability of our results with those of DE-REACH. While DE-REACH required counselors to have three years of healthcare training (e.g., as occupational therapists or nurses) and professional experience in dementia care, the latter was not required for our npMPS. In our study, the training time was only 12 h, and the counseling time was 5 h per case, both by protocol and in practice, which was considerably shorter than in the DE-REACH intervention (over 10.5 h). With these resource-efficient adaptations, our intervention understandably could not slow the increase in ZBI to the same extent as the DE-REACH intervention but was still superior to usual care while receiving exceptionally high ratings for feasibility.

Our findings align well with current evidence on interventions for informal caregivers of people with dementia. A recent meta-analysis of 31 randomized controlled trials (RCTs) found that multi-component interventions—delivered directly or remotely (phone, online) and combining elements such as psychoeducation or therapy, skills training, professional or peer support, case management, exercise, memory clinic visits, and meditation/mindfulness—improved subjective well-being, mood, and anxiety, though caregiver burden improved only with direct contact [38]. Another meta-analysis of 85 RCTs identified case management, psychoeducation, and multi-component approaches as effective in reducing caregiver burden [39]. Finally, a recent scoping review of 26 studies showed that psychoeducational and cognitive-behavioral interventions enhance self-esteem, coping, and problem-solving among caregivers [40]. Our intervention incorporated these evidence-based facets, including a multi-component approach and psychoeducational and cognitive-behavioral elements.

### Task shifting and sharing in general practices

Given the shortage of healthcare professionals, informal caregivers play a crucial role in caring for people with dementia, yet they themselves need support in this demanding function. However, it is not clear who is best placed to provide this support and coordinate care. While npMPS in general practices are predestined to take on this new role, several barriers need to be considered. In addition to the need for structural changes in general practice, e.g., regarding reimbursement policies (see also below), unclear role definitions and competitive behavior among practice team members may hinder the implementation of an appropriate new care model for informal caregivers of people with dementia [41]. Given the heterogeneity in education and competencies of the npMPS, our pragmatic approach sought to address the lack of role clarity by providing a straightforward and practice-oriented training tailored to the practice team setting. It is possibly also due to this approach that the majority of GPs and npMPS in our study viewed the npMPS-led intervention as adding value to their practice and were motivated to continue the intervention beyond the study period. This indicates that Swiss GPs and npMPS are open to implementing new models of care if the aforementioned barriers can be overcome. Our approach could also serve as a model for task shifting and task sharing in general practice across other healthcare systems, irrespective of npMPS role configurations, by overcoming unclear role definitions and related barriers.

The informal caregivers, as recipients of the service, also responded positively to the task shift, expressing high levels of agreement with the npMPS's competence. The role of the GPs as involved participants was considered crucial, suggesting that shared responsibility within the practice team, rather than a complete task shift from GPs to npMPS, is optimal, a success factor also highlighted in the review by Busca et al. [41].

Interestingly and surprisingly, only half of the informal caregivers found the home visit setting of the supportive counseling important, while a substantial proportion preferred meetings at the practice premises. A possible explanation is that they could talk more openly about their problems and feelings in the practice setting than at home, possibly with the person with dementia in the same room. This, together with the opportunity to do something on their own, seems to counterbalance the task of organizing a substitute caregiver during the encounter.

### Implications for practice

We have proposed a pragmatic model that takes into account the limited (time) resources of general practice teams and their familiarity with the informal caregivers' needs. To overcome the primary barriers to implementing our intervention, structural changes at the health policy and healthcare system level are essential. These changes should include adequate reimbursement for npMPS and strategies to address the shortage of both npMPS and GPs. A recent study has also highlighted substantial variability in the role descriptions, education, supervision, and support of non-physician professionals working in advanced clinical practice, underscoring the need for standardization and governance in these professions [42]. On the organizational level within practices, it is important to clarify the roles and responsibilities of npMPS when taking on new tasks, as this positively impacts team dynamics and care outcomes, including improvements in patient-related experience and outcome measures [43].

### Implications for research

The literature describes different types of caregiver-patient dyads that differ greatly in stressors and therefore require targeted support strategies [44]. Future research could focus on the effects of our intervention on such dyads. Further studies should also clarify the contribution of the different elements of our intervention to its overall effect, for example, the psychological relief of simply being able to talk about the difficulties of caring for a relative, self-management skills, support in dealing with the care recipient's behavioral symptoms, or help in coordinating care. Finally, as our study differed from DE-REACH in terms of both resources required and effectiveness, the cost-effectiveness of the different interventions should also be investigated to find an optimal balance between resources, cost, and effectiveness.

### Strengths and limitations

The results obtained in our study depended on the caregivers' motivation as well as the individual skills of the npMPS and GPs, which reflects reality quite well and suggests that the stabilizing effect on informal caregiver burden should also be achievable outside the study setting—at least in Switzerland and healthcare systems with comparable reimbursement structures, workforce composition, and primary care organization.

Because there was a great deal of variability in the caregivers' circumstances and therefore in the recommended measures, we were only able to demonstrate the feasibility and effectiveness of the intervention as a whole, but not to determine the precise effects of the individual elements of the intervention. The heterogeneity of recommended measures may also impede study reproducibility. Due to the available resources, we did not conduct the study as a randomized trial with an internal control group, which carries a risk of unmeasured confounding and limits causal inference, and since the external ZBI increase used as comparator does not account for individual baselines, we did not adjust our own analysis accordingly. The baseline ZBI difference between all included caregivers and those providing data at T5 might, despite comparable quality of life, indicate attrition (e.g., due to institutionalization of care recipients) and resulting survival bias. Finally, the sample size calculated for the primary outcome was insufficient to identify associations with caregiver characteristics with adequate statistical power and may therefore have led to false-negative results for secondary outcomes.

## Conclusion

Our results indicate that our supportive intervention for informal caregivers of patients with dementia, led by general practice teams, may help to mitigate the burden of informal caregivers. The collaborative approach with task-sharing in general practices proved effective, feasible, and well-accepted by medical professionals and informal caregivers alike, thus offering a resource-efficient model for supporting informal dementia caregivers in routine primary and geriatric care.

## Supplementary Information

Below is the link to the electronic supplementary material.

Supplementary file1 (DOCX 60 KB)

Supplementary file2 (DOCX 122 KB)

## Data Availability

De-identified patient data, the R script used for analysis, and all study materials will be made available by the corresponding author upon reasonable request.

## Abbreviations

APN: Advanced practice nurse
DE-REACH: German adaptation of REACH II
GP: General practitioner
MPA: Medical practice assistant
MPC: Medical practice coordinator
npMPS: Non-physician medical practice staff
QoL: Quality of life
REACH: Resources for Enhancing Alzheimer's Caregiver Health
REDCap: Research Electronic Data Capture
VAS: Visual analogue scale (of the EQ-5D-5L instrument).
WIS: Weighted index score (of the EQ-5D-5L instrument)
ZBI: Zarit Burden Interview

## References

[1] Prince M, Bryce R, Albanese E, Wimo A, Ribeiro W, Ferri CP (2013) The global prevalence of dementia: a systematic review and metaanalysis. Alzheimers Dement 9(1):63-75.e2 23305823 10.1016/j.jalz.2012.11.007

[2] Alzheimer Schweiz (2023) Demenz in der Schweiz 2023: Zahlen und Fakten. Alzheimer Schweiz, Bern

[3] Alzheimer’s Association (2022) 2022 Alzheimer’s disease facts and figures. Alzheimers Dement 18(4):700–789 35289055 10.1002/alz.12638

[4] Fratiglioni L, Launer LJ, Andersen K, Breteler MM, Copeland JR, Dartigues JF et al (2000) Incidence of dementia and major subtypes in Europe A collaborative study of population-based cohorts Neurologic. Diseases in the elderly research group. Neurology 54(11 Suppl 5):S10–S15 10854355

[5] Pudelewicz A, Talarska D, Bączyk G (2019) Burden of caregivers of patients with Alzheimer’s disease. Scand J Caring Sci 33(2):336–341 30378698 10.1111/scs.12626

[6] Alzheimer Schweiz (2014) Angehörige von Menschen mit Demenz geben Auskunft. Alzheimer Schweiz, Yverdon-les-Bains

[7] Bannerot F, Leocadie MC, Rothan-Tondeur M (2019) Determining factors in the use of respite services by caregivers of patients with dementia: qualitative research using focus groups. Sante Publique 31(2):277–286 33305931 10.3917/spub.192.0277

[8] König M, Wettstein A (2002) Caring for relatives with dementia: willingness-to-pay for a reduction in caregiver’s burden. Expert Rev Pharmacoecon Outcomes Res 2(6):535–547 19807478 10.1586/14737167.2.6.535

[9] Adelman RD, Tmanova LL, Delgado D, Dion S, Lachs MS (2014) Caregiver burden: a clinical review. JAMA 311(10):1052–1060 24618967 10.1001/jama.2014.304

[10] Liu Z, Heffernan C, Tan J (2020) Caregiver burden: a concept analysis. Int J Nurs Sci 7(4):438–445 33195757 10.1016/j.ijnss.2020.07.012 PMC7644552

[11] Blom M, Duijnstee M, Schnepp W, Witthaut D, Emmrich D (1996) Wie soll ich das nur aushalten? Mit dem Pflegekompass die Belastung pflegender Angehöriger einschätzen. Huber, Bern

[12] Chiao CY, Wu HS, Hsiao CY (2015) Caregiver burden for informal caregivers of patients with dementia: a systematic review. Int Nurs Rev 62(3):340–350 26058542 10.1111/inr.12194

[13] Pinquart M, Sörensen S (2003) Associations of stressors and uplifts of caregiving with caregiver burden and depressive mood: a meta-analysis. J Gerontol B Psychol Sci Soc Sci 58(2):112–128 10.1093/geronb/58.2.p112 12646594

[14] Sanders S (2005) Is the glass half empty or full? Reflections on strain and gain in cargivers of individuals with Alzheimer’s disease. Soc Work Health Care 40(3):57–73 15837668 10.1300/J010v40n03_04

[15] Cohen CA, Colantonio A, Vernich L (2002) Positive aspects of caregiving: rounding out the caregiver experience. Int J Geriatr Psychiatry 17(2):184–188 11813283 10.1002/gps.561

[16] Brodaty H, Donkin M (2009) Family caregivers of people with dementia. Dialogues Clin Neurosci 11(2):217–228 19585957 10.31887/DCNS.2009.11.2/hbrodaty PMC3181916

[17] Ma M, Dorstyn D, Ward L, Prentice S (2018) Alzheimer’s disease and caregiving: a meta-analytic review comparing the mental health of primary carers to controls. Aging Ment Health 22(11):1395–1405 28871796 10.1080/13607863.2017.1370689

[18] Sallim AB, Sayampanathan AA, Cuttilan A, Ho RC-M (2015) Prevalence of mental health disorders among caregivers of patients with Alzheimer disease. J Am Med Dir Assoc 16(12):1034–1041 26593303 10.1016/j.jamda.2015.09.007

[19] Sheehan OC, Haley WE, Howard VJ, Huang J, Rhodes JD, Roth DL (2021) Stress, burden, and well-being in dementia and nondementia caregivers: insights from the caregiving transitions study. Gerontologist 61(5):670–679 32816014 10.1093/geront/gnaa108 PMC8276607

[20] Gitlin LN, Marx K, Stanley IH, Hodgson N (2015) Translating evidence-based dementia caregiving interventions into practice: state-of-the-science and next steps. Gerontologist 55(2):210–226 26035597 10.1093/geront/gnu123 PMC4542834

[21] Wisniewski SR, Belle SH, Coon DW, Marcus SM, Ory MG, Burgio LD et al (2003) The resources for enhancing Alzheimer’s caregiver health (REACH): project design and baseline characteristics. Psychol Aging 18(3):375–384 14518801 10.1037/0882-7974.18.3.375 PMC2577188

[22] Vandepitte S, Van Den Noortgate N, Putman K, Verhaeghe S, Verdonck C, Annemans L (2016) Effectiveness of respite care in supporting informal caregivers of persons with dementia: a systematic review. Int J Geriatr Psychiatry 31(12):1277–1288 27245986 10.1002/gps.4504

[23] Weinbrecht A, Rieckmann N, Renneberg B (2016) Acceptance and efficacy of interventions for family caregivers of elderly persons with a mental disorder: a meta-analysis. Int Psychogeriatr 28(10):1615–1629 27268305 10.1017/S1041610216000806

[24] Gilhooly KJ, Gilhooly ML, Sullivan MP, McIntyre A, Wilson L, Harding E et al (2016) A meta-review of stress, coping and interventions in dementia and dementia caregiving. BMC Geriatr 16:106 27193287 10.1186/s12877-016-0280-8 PMC4872341

[25] Schulz R (2000) Handbook on dementia caregiving: Evidence-based interventions for family caregivers. Springer Publishing Company, New York

[26] Belle SH, Burgio L, Burns R, Coon D, Czaja SJ, Gallagher-Thompson D et al (2006) Enhancing the quality of life of dementia caregivers from different ethnic or racial groups: a randomized, controlled trial. Ann Intern Med 145(10):727–738 17116917 10.7326/0003-4819-145-10-200611210-00005 PMC2585490

[27] Berwig M, Heinrich S, Spahlholz J, Hallensleben N, Brähler E, Gertz HJ (2017) Individualized support for informal caregivers of people with dementia - effectiveness of the German adaptation of REACH II. BMC Geriatr 17(1):286 29233097 10.1186/s12877-017-0678-y PMC5728045

[28] Phillips WR, Sturgiss E, Glasziou P, Olde Hartman TC, Orkin AM, Prathivadi P et al (2023) Improving the reporting of primary care research: consensus reporting items for studies in primary care-the CRISP statement. Ann Fam Med 21(6):549–555 37788942 10.1370/afm.3029 PMC10681700

[29] Rička R, Kessler C, Bally K, Luchsinger P, von Wartburg L (2021) Entlastungsbedarf von betreuenden Angehörigen in der ärztlichen Praxis erfassen. Prim Hosp Care Allg Inn Med 21(02):60–63

[30] Smidt A, Balandin S, Sigafoos J, Reed VA (2009) The Kirkpatrick model: a useful tool for evaluating training outcomes. J Intellect Dev Disabil 34(3):266–274 19681007 10.1080/13668250903093125

[31] Braun M, Scholz U, Hornung R, Martin M (2010) Caregiver burden with dementia patients. A validation study of the German language version of the Zarit Burden Interview. Z Gerontol Geriatr 43(2):111–119 20204383 10.1007/s00391-010-0097-6

[32] Kühnel MB, Ramsenthaler C, Bausewein C, Fegg M, Hodiamont F (2020) Validation of two short versions of the Zarit Burden Interview in the palliative care setting: a questionnaire to assess the burden of informal caregivers. Support Care Cancer 28(11):5185–5193 32060707 10.1007/s00520-019-05288-w PMC7546983

[33] Schreiner AS, Morimoto T, Arai Y, Zarit S (2006) Assessing family caregiver’s mental health using a statistically derived cut-off score for the Zarit Burden Interview. Aging Ment Health 10(2):107–111 16517485 10.1080/13607860500312142

[34] EuroQol Research Foundation (2019) EQ-5D-5L user guide. EuroQol Research Foundation, Rotterdam

[35] Harris PA, Taylor R, Thielke R, Payne J, Gonzalez N, Conde JG (2009) Research electronic data capture (REDCap) - a metadata-driven methodology and workflow process for providing translational research informatics support. J Biomed Inform 42(2):377–381 18929686 10.1016/j.jbi.2008.08.010 PMC2700030

[36] R Core Team (2024) R: a language and environment for statistical computing. Version 4.4.0. Vienna: R foundation for statistical computing.

[37] VERBI Software. MAXQDA Software for qualitative data analysis. Berlin: VERBI Software; 1989–2024.

[38] He J, Wang J, Zhong H, Guan C (2022) The effectiveness of multi-component interventions on the positive and negative aspects of well-being among informal caregivers of people with dementia: a systematic review and meta-analysis. Int J Environ Res Public Health 19(12):6973 35742220 10.3390/ijerph19126973 PMC9222573

[39] Sun Y, Ji M, Leng M, Li X, Zhang X, Wang Z (2022) Comparative efficacy of 11 non-pharmacological interventions on depression, anxiety, quality of life, and caregiver burden for informal caregivers of people with dementia: a systematic review and network meta-analysis. Int J Nurs Stud 129:104204 35247788 10.1016/j.ijnurstu.2022.104204

[40] Encinas-Monge C, Hidalgo-Fuentes S, Cejalvo E, Martí-Vilar M (2024) Interventions to relieve the burden on informal caregivers of older people with dementia: a scoping review. Nurs Rep 14(3):2535–2549 39311195 10.3390/nursrep14030187 PMC11417853

[41] Busca E, Savatteri A, Calafato TL, Mazzoleni B, Barisone M, Dal Molin A (2021) Barriers and facilitators to the implementation of nurse’s role in primary care settings: an integrative review. BMC Nurs 20(1):171 34530813 10.1186/s12912-021-00696-y PMC8444166

[42] Fothergill LJ, Al-Oraibi A, Houdmont J, Conway J, Evans C, Timmons S et al (2022) Nationwide evaluation of the advanced clinical practitioner role in England: a cross-sectional survey. BMJ Open 12(1):e055475 34987045 10.1136/bmjopen-2021-055475 PMC8734004

[43] Kilpatrick K, Tchouaket E, Fernandez N, Jabbour M, Dubois CA, Paquette L et al (2021) Patient and family views of team functioning in primary healthcare teams with nurse practitioners: a survey of patient-reported experience and outcomes. BMC Fam Pract 22(1):76 33866963 10.1186/s12875-021-01406-y PMC8054435

[44] Wiegelmann H, Wolf-Ostermann K, Brannath W, Arzideh F, Dreyer J, Thyrian R et al (2021) Sociodemographic aspects and health care-related outcomes: a latent class analysis of informal dementia care dyads. BMC Health Serv Res 21(1):727 34301241 10.1186/s12913-021-06708-6 PMC8299572

