# Supplementary material for: Effectiveness, feasibility, and acceptance of a general practice-based supportive intervention for dementia caregivers: the AD HOC trial
Source: Eur Geriatr Med. 2026 Feb 25;17(3):1489–500. doi: 10.1007/s41999-026-01437-7 (PMC13309503; doi:10.1007/s41999-026-01437-7)
Supplement: Supplementary file 1 — Supplementary file1 (DOCX 60 KB) [file 41999_2026_1437_MOESM1_ESM.docx]

**SUPPLEMENTARY MATERIAL**

**Publication**:

Bopp G, Grischott T, Pichierri G, Greindl S, Gysin S, Senn O, Rosemann T, Neuner-Jehle S. Effectiveness, Feasibility, and Acceptance of a General Practice-Based Supportive Intervention for Dementia Caregivers: The AD HOC Trial. Eur Geriatr Med. 2026. doi: 10.1007/s41999-026-01437-7.

**Corresponding Author**:

Thomas Grischott, MD MSc, Institute of Primary Care, University Hospital Zurich, University of Zurich, Sonneggstrasse 6, 8091 Zurich, Switzerland

[thomas.grischott@usz.ch](mailto:thomas.grischott@usz.ch)

**Supplementary Table 1** Outline and content of the npMPS training*. In the first training session, our dementia experts provided the participating npMPS with basic information about dementia, including the pathological mechanism, behavioral changes and classic problems that occur as the disease progresses. The npMPS were also familiarized with the study protocol. In the second session, the npMPS were trained on how to best connect with caregivers and how to use our conversation guide. Finally, the third session provided an opportunity for the npMPS to share their experiences in supporting their caregivers, receive input from our experts, and discuss problem cases.*

| **Session**  **number** | **Session theme** | **Content/topics** | **Course format** | **Duration** |
| --- | --- | --- | --- | --- |
| 1 | Knowledge transfer | - Information on dementia - Overview of study procedures and documents | In-person | 4h |
| 2 | Study task-oriented training | - Conversation techniques - Initial contact setup - Planning home visits - Handling challenging situations for npMPS   and informal caregivers | In-person,  role play | 4h |
| 3 | Tracking and support | - Case presentation - Experience exchange - Clarification of ambiguities - Course evaluation | In-person | 4h |

*Abbreviation: npMPS: non-physician medical practice staff.*

**Supplementary Table 2** Overview of the use of the Kirkpatrick model to evaluate the npMPS training. *The satisfaction of the npMPS with the training was measured using questionnaires. Their learning success in terms of knowledge and skills, in particular the correct application of the learned interview techniques, was evaluated by the instructing experts in exercises and case discussions. At the end of the 3-month period, the participants met to discuss their experiences and to verify, under the guidance of the experts, that they were able to apply what they had learned. The impact of the training on caregiver burden and quality of life was assessed through feedback questionnaires, completed by the caregivers at the end of the study.*

| **Level** | **Outcome/key question(s)** | **Measurement method** |
| --- | --- | --- |
| **Reaction** | - How did participants react to the training? - Did they find it engaging and useful? | Questionnaires |
| **Learning** | - Has knowledge increased? - Can participants effectively approach a case? - Can they set up an interview? | Peers/experts observation during case vignette management to assess learning application |
| **Behavior** | - Can participants manage the informal caregiver’s case? | Experience exchange  3 months after training |
| **Results** | - Did the intervention impact the caregiver’s burden and quality of life? | Intervention effectiveness results and questionnaires |

*Abbreviation: npMPS: non-physician medical practice staff.*

**Supplementary Table 3** Overview of the conversation guide used in the first home visit. *During the first home visit, the npMPS used the conversation guide to get an idea of the individual situation of the informal caregivers and the people with dementia they care for, including specific challenges, existing resources, and support needs. Support actions and services were then planned based on the results of this conversation.*

| **Section** | **Items** | **Answer format** |
| --- | --- | --- |
| **Opening the conversation** | - Conveying empathy, understanding, and appreciation - Clarifying the purpose of the conversation   ('to help you feel better') | Open |
| **Basic information about the informal caregiver** | - Why and since when is the caregiver involved? - Support services already utilized for the person with dementia and for the caregiver themselves,   including any additional informal support   - Occupation, duties, and additional caregiving responsibilities or other obligations - Caregiver’s personal health condition/issues | Partly structured (multiple choice) and open |
| **Basic information about the person with dementia** | - Demographic data - Type and severity of dementia - Other health conditions - Relationship to the informal caregiver and living situation | Structured (multiple choice) and open |
| **Exploration of care and support tasks** | - Caregiver’s tasks:   Being present, organizational and administrative tasks, instrumental assistance, personal care, medical care   - Behavior of the person with dementia:   Delusions, sensory distortions, agitation, depression, irritability, anxiety, elevated mood, lack of drive and interest, disinhibition, impulsivity, mood swings, restlessness, nighttime behavior, appetite, eating habits   - Self-assessment of the informal caregiver:   Fatigue, feeling abandoned, difficulty delegating responsibility, restlessness, overwhelm | Single choice (per item): not vs. some-what vs. very burden-some (or not specified)  Two single choice answers (per item): applicable/concerning or not, and not vs. somewhat vs. very burdensome  Single choice (per item): not vs. partially vs. fully applicable |
| **Resources/support** | - Adequate opportunities and time for retreat - Time for social contacts and relationships - Ability to reconcile all tasks - Financial security - Sources of strength and endurance? | Single choice (per item): not vs. partially vs. fully applicable  Open |
| **Concluding the conversation** | - Greatest burden, greatest wish? - Decision/explanation of further steps | Open |

*Abbreviation: npMPS: non-physician medical practice staff.*

**Supplementary Table 4** Associations of ZBI score increases and quality of life (WIS and VAS) changes with caregiver and npMPS characteristics

### ZBI score increases over 6 and 9 months

#==========================================

# zbi_d5: ZBI score increase from T0 to T5 (6 months)

# zbi_d6: ZBI score increase from T0 to T6 (9 months)

> summary(pool(with(imp, lmer(zbi_d5 ~ ... + (1 | mpfp_id)))))

# term est SE t df p

# (Intercept) -6.79544 21.16247 -0.321108 26.726 0.75062

# caregiver_sexmale -0.89156 4.46868 -0.199514 25.575 0.84343

# caregiver_age 0.23129 0.24591 0.940548 22.301 0.35701

# caregiver_educationmatura -0.50953 4.77554 -0.106697 25.346 0.91586

# caregiver_educationuniversity 7.77170 5.44327 1.427763 23.443 0.16654

# caregiver_civil_statusmarried -19.71895 15.39501 -1.280866 20.862 0.21429

# caregiver_civil_statusregistered -7.80538 17.71279 -0.440663 24.078 0.66338

# caregiver_civil_statussingle -13.30477 24.28313 -0.547901 21.645 0.58936

# caregiver_nationalityswiss 6.97153 9.07911 0.767865 26.269 0.44941

# caregiver_relationfamily -1.94465 16.56187 -0.117417 15.155 0.90807

# caregiver_relationspouse -2.29653 14.52130 -0.158149 18.890 0.87601

# caregiver_housingsolitarily 9.00204 18.06664 0.498269 27.292 0.62228

# caregiver_housingwithothers 0.58597 14.37392 0.040766 28.024 0.96777

# caregiver_housingwithpatient -5.79007 19.52056 -0.296614 22.987 0.76942

# npmps_age 0.18750 0.35178 0.533025 28.241 0.59818

# npmps_educationmpa 4.30928 10.22880 0.421289 28.294 0.67672

# npmps_educationmpk -1.03311 9.54639 -0.108220 27.611 0.91460

# npmps_educationother -8.40561 12.03352 -0.698516 29.037 0.49041

# npmps_experience 0.10773 0.39519 0.272610 28.596 0.78711

# npmps_practicepair 1.13417 8.32219 0.136283 28.746 0.89254

# npmps_practicesolo -18.12080 14.05016 -1.289722 23.712 0.20958

> summary(pool(with(imp, lmer(zbi_d6 ~ ... + (1 | mpfp_id)))))

# term est SE t df p

# (Intercept) 3.30890 22.44580 0.147417 22.572 0.88411

# caregiver_sexmale 3.10120 4.53369 0.684034 26.723 0.49984

# caregiver_age 0.07186 0.25684 0.279800 22.403 0.78219

# caregiver_educationmatura 3.35072 5.02351 0.667008 24.185 0.51108

# caregiver_educationuniversity 6.75721 5.55449 1.216529 25.612 0.23487

# caregiver_civil_statusmarried -2.72864 17.37352 -0.157057 16.077 0.87715

# caregiver_civil_statusregistered 3.70430 19.04015 0.194552 21.878 0.84753

# caregiver_civil_statussingle -2.82542 24.40712 -0.115762 20.483 0.90896

# caregiver_nationalityswiss -9.75922 10.45675 -0.933294 20.792 0.36138

# caregiver_relationfamily -1.59109 16.39530 -0.097045 17.374 0.92380

# caregiver_relationspouse -1.15168 16.57238 -0.069494 15.534 0.94548

# caregiver_housingsolitarily -33.26820 20.62342 -1.613127 21.868 **0.12105**

# caregiver_housingwithothers -4.11284 17.54234 -0.234452 18.749 0.81717

# caregiver_housingwithpatient -9.98742 21.87117 -0.456647 18.217 0.65332

# npmps_age 0.18216 0.33339 0.546402 27.298 0.58922

# npmps_educationmpa 8.77959 9.56174 0.918199 28.778 0.36614

# npmps_educationmpk 2.57327 8.89526 0.289285 28.016 0.77449

# npmps_educationother -2.12739 11.13911 -0.190984 28.267 0.84990

# npmps_experience -0.18289 0.36953 -0.494919 27.489 0.62459

# npmps_practicepair 5.76041 7.60946 0.757006 28.743 0.45520

# npmps_practicesolo -7.59364 13.87703 -0.547208 22.558 0.58960

### WIS changes over 6 and 9 months

#==================================

# wis_d5: WIS change from T0 to T5 (6 months)

# wis_d6: WIS change from T0 to T6 (9 months)

> summary(pool(with(imp, lmer(wis_d5 ~ ... + (1 | mpfp_id)))))

# term est SE t df p

# (Intercept) -0.124680 0.180796 -0.689619 26.695 0.49638

# caregiver_sexmale 0.055152 0.043172 1.277511 24.206 0.21353

# caregiver_age 0.003175 0.002510 1.264823 22.121 0.21909

# caregiver_educationmatura -0.003209 0.044517 -0.072095 26.630 0.94306

# caregiver_educationuniversity 0.034559 0.057802 0.597886 24.678 0.55536

# caregiver_civil_statusmarried 0.021909 0.151876 0.144261 16.263 0.88706

# caregiver_civil_statusregistered 0.073028 0.179964 0.405791 21.123 0.68897

# caregiver_civil_statussingle 0.072739 0.220776 0.329469 16.376 0.74597

# caregiver_nationalityswiss 0.041595 0.100989 0.411882 21.854 0.68443

# caregiver_relationfamily -0.083561 0.155845 -0.536185 16.862 0.59883

# caregiver_relationspouse -0.063150 0.142401 -0.443464 18.615 0.66254

# caregiver_housingsolitarily -0.183991 0.195627 -0.940518 23.561 0.35649

# caregiver_housingwithothers -0.065748 0.140194 -0.468984 27.571 0.64276

# caregiver_housingwithpatient -0.115669 0.196974 -0.587231 20.950 0.56332

# npmps_age -0.002267 0.002561 -0.885084 21.012 0.38612

# npmps_educationmpa 0.048782 0.075282 0.647995 20.767 0.52408

# npmps_educationmpk 0.013072 0.071688 0.182349 20.347 0.85711

# npmps_educationother 0.003541 0.076028 0.046580 22.875 0.96325

# npmps_experience 0.000468 0.002597 0.180341 21.622 0.85856

# npmps_practicepair -0.039662 0.054907 -0.722361 20.873 0.47808

# npmps_practicesolo 0.018005 0.147705 0.121904 12.046 0.90498

> summary(pool(with(imp, lmer(wis_d6 ~ ... + (1 | mpfp_id)))))

# term est SE t df p

# (Intercept) -0.034910 0.261864 -0.133313 22.597 0.89512

# caregiver_sexmale 0.036100 0.056670 0.637024 25.519 0.52978

# caregiver_age -0.000208 0.003263 -0.063836 23.161 0.94964

# caregiver_educationmatura 0.005779 0.061729 0.093623 24.470 0.92617

# caregiver_educationuniversity 0.022699 0.074393 0.305123 25.785 0.76272

# caregiver_civil_statusmarried -0.010235 0.199640 -0.051267 17.546 0.95969

# caregiver_civil_statusregistered 0.043918 0.234418 0.187353 22.710 0.85304

# caregiver_civil_statussingle 0.038485 0.269095 0.143018 21.156 0.88762

# caregiver_nationalityswiss -0.046147 0.141817 -0.325399 19.134 0.74841

# caregiver_relationfamily -0.023916 0.188083 -0.127159 21.296 0.90000

# caregiver_relationspouse -0.018025 0.169066 -0.106619 24.491 0.91595

# caregiver_housingsolitarily -0.017128 0.259342 -0.066045 23.570 0.94789

# caregiver_housingwithothers 0.092887 0.205936 0.451047 22.102 0.65634

# caregiver_housingwithpatient 0.061260 0.274314 0.223321 19.714 0.82558

# npmps_age 0.000469 0.003397 0.138204 24.765 0.89119

# npmps_educationmpa 0.023698 0.101450 0.233600 23.917 0.81728

# npmps_educationmpk 0.033599 0.094388 0.355974 23.980 0.72497

# npmps_educationother 0.108346 0.103319 1.048652 26.886 0.30367

# npmps_experience -0.002036 0.003499 -0.582019 25.869 0.56559

# npmps_practicepair -0.007560 0.072977 -0.103603 25.684 0.91828

# npmps_practicesolo -0.005040 0.165822 -0.030396 18.933 0.97606

### VAS change over 6 and 9 months

#=================================

# vas_d5: VAS change from T0 to T5 (6 months)

# vas_d6: VAS change from T0 to T6 (9 months)

> summary(pool(with(imp, lmer(vas_d5 ~ ... + (1 | mpfp_id)))))

# term est SE t df p

# (Intercept) -11.1174 33.474967 -0.332113 25.547 0.74251

# caregiver_sexmale -1.8502 8.207827 -0.225427 20.687 0.82385

# caregiver_age 0.6036 0.430988 1.400526 22.809 0.17481

# caregiver_educationmatura -5.2433 8.472801 -0.618843 22.485 0.54223

# caregiver_educationuniversity 2.6211 10.794289 0.242831 19.586 0.81066

# caregiver_civil_statusmarried -14.2636 25.806736 -0.552709 18.829 0.58696

# caregiver_civil_statusregistered -7.3161 30.040929 -0.243538 24.242 0.80963

# caregiver_civil_statussingle -5.1271 39.326376 -0.130374 18.319 0.89769

# caregiver_nationalityswiss -13.5160 17.661891 -0.765265 21.059 0.45260

# caregiver_relationfamily -8.5669 28.298839 -0.302731 15.818 0.76603

# caregiver_relationspouse -2.8350 24.828269 -0.114185 19.429 0.91026

# caregiver_housingsolitarily -23.0949 33.879984 -0.681668 23.495 0.50211

# caregiver_housingwithothers -6.1452 24.891122 -0.246885 27.299 0.80684

# caregiver_housingwithpatient -8.9317 33.771008 -0.264480 21.950 0.79387

# npmps_age 0.1967 0.493503 0.398731 23.816 0.69364

# npmps_educationmpa 10.1045 14.373603 0.702990 24.377 0.48872

# npmps_educationmpk 9.1907 13.590644 0.676256 23.253 0.50554

# npmps_educationother 6.5240 15.520224 0.420360 25.717 0.67771

# npmps_experience -0.5356 0.552496 -0.969580 21.467 0.34305

# npmps_practicepair -8.8399 11.484769 -0.769712 21.772 0.44974

# npmps_practicesolo 0.3618 24.235014 0.014932 16.099 0.98826

> summary(pool(with(imp, lmer(vas_d6 ~ ... + (1 | mpfp_id)))))

# term est SE t df p

# (Intercept) -14.41784 32.47241 -0.444002 22.339 0.66131

# caregiver_sexmale 10.08977 7.12136 1.416830 24.591 0.16907

# caregiver_age 0.08679 0.42604 0.203722 20.777 0.84055

# caregiver_educationmatura -8.12894 7.61006 -1.068183 24.938 0.29566

# caregiver_educationuniversity 3.26343 9.29437 0.351118 26.220 0.72830

# caregiver_civil_statusmarried -2.04688 22.94794 -0.089196 20.788 0.92977

# caregiver_civil_statusregistered -1.22665 28.52436 -0.043003 23.859 0.96605

# caregiver_civil_statussingle -1.44540 34.11252 -0.042371 19.947 0.96662

# caregiver_nationalityswiss -15.60050 16.39244 -0.951688 23.095 0.35110

# caregiver_relationfamily -0.23821 25.99810 -0.009162 17.053 0.99279

# caregiver_relationspouse 3.28731 25.44755 0.129179 16.169 0.89880

# caregiver_housingsolitarily 4.86833 34.92574 0.139391 19.592 0.89056

# caregiver_housingwithothers 22.05522 26.46210 0.833464 20.348 0.41425

# caregiver_housingwithpatient 25.49050 33.40104 0.763164 19.624 0.45444

# npmps_age 0.27131 0.40347 0.672455 24.426 0.50760

# npmps_educationmpa 6.42389 11.62398 0.552641 25.612 0.58530

# npmps_educationmpk 0.02716 11.16059 0.002433 24.289 0.99807

# npmps_educationother 8.75047 11.97789 0.730552 26.925 0.47136

# npmps_experience -0.53128 0.45283 -1.173246 19.849 0.25458

# npmps_practicepair -6.74903 8.63764 -0.781350 24.690 0.44202

# npmps_practicesolo 8.60461 19.75731 0.435515 20.172 0.66781

**Supplementary Tables 5-7** Process evaluation results among GPs, npMPS, and informal caregivers

**Supplementary Table 5** Assessment and acceptance of the new role of the npMPS in caregiver support by the GPs in participating practices

*Abbreviations: npMPS: non-physician medical practice staff; GP: general practitioner.*

| **Questions for GPs** | *n* | Dis-agree (%) | Rather dis-agree  (%) | Neutral (%) | Rather agree (%) | Agree (%) |
| --- | --- | --- | --- | --- | --- | --- |
| Distinguishing my own role from the new npMPS role was challenging. | 17 | 76.5 | 17.6 | 5.9 | 0 | 0 |
| The new npMPS role adds value to the practice. | 16 | 0 | 0 | 6.3 | 25.0 | 68.8 |
| I would like to establish the new npMPS role permanently in my practice. | 17 | 5.9 | 17.6 | 5.9 | 17.6 | 52.9 |
| The new npMPS role was well received by the practice team. | 17 | 0 | 5.9 | 0 | 29.4 | 64.7 |
| As a physician, I find the new npMPS role a welcome relief. | 17 | 0 | 5.9 | 11.8 | 11.8 | 70.6 |
| *For GPs who attended the training:*  The training prepared me well for my study tasks (e.g., supervising the npMPS). | 1 | 0 | 0 | 0 | 0 | 100 |
| Discussions with the npMPS helped me better understand their new role. | 17 | 0 | 0 | 0 | 47.1 | 52.9 |
| Overall, I find the project’s approach practical. | 17 | 0 | 0 | 5.9 | 23.5 | 70.6 |

**Supplementary Table 6** Acceptance and feasibility of the intervention among the npMPS

| **Questions for npMPS** | *n* | Dis-agree (%) | Rather dis-agree (%) | Neutral (%) | Rather agree (%) | Agree (%) |
| --- | --- | --- | --- | --- | --- | --- |
| Distinguishing my role from that of other professionals was difficult. | 20 | 50.0 | 20.0 | 25.0 | 5.0 | 0 |
| Counseling was more challenging for me than administrative tasks. | 20 | 45.0 | 40.0 | 10.0 | 5.0 | 0 |
| My new role has added significant personal value. | 19 | 5.3 | 15.8 | 15.8 | 26.3 | 36.8 |
| I believe my new role benefits the informal caregivers I have counseled. | 20 | 0 | 5.0 | 25.0 | 45.0 | 25.0 |
| I think my new role provides value to the GP  (e.g., time relief). | 20 | 5.0 | 15.0 | 15.0 | 35.0 | 30.0 |
| I would like to continue in this new role. | 18 | 0 | 5.6 | 22.2 | 16.7 | 55.6 |
| I felt my contact with other professional supporters was welcomed. | 18 | 0 | 0 | 11.1 | 38.9 | 50.0 |
| The training prepared me well for my tasks. | 20 | 0 | 0 | 20.0 | 25.0 | 55.0 |
| The conversation guide for the initial counseling was helpful. | 20 | 0 | 0 | 0 | 45.0 | 55.0 |
| The sequence of study steps was easy to follow. | 20 | 0 | 5.0 | 10.0 | 35.0 | 50.0 |
| Consulting with the GP was easily accessible. | 20 | 0 | 0 | 20.0 | 10.0 | 70.0 |
| The progress sheet (problems, actions, implementation) was useful. | 20 | 0 | 5.0 | 5.0 | 40.0 | 50.0 |
| I had enough time for the counseling sessions. | 20 | 0 | 5.0 | 5.0 | 5.0 | 85.0 |
| I had enough time to plan actions between visits. | 20 | 0 | 5.0 | 10.0 | 10.0 | 75.0 |
| I had enough time to consult with the GP. | 19 | 0 | 5.3 | 10.5 | 5.3 | 78.9 |
| Supervision improved my effectiveness in subsequent consultations. | 18 | 5.6 | 16.7 | 16.7 | 44.4 | 16.7 |
| The situation with family caregivers at home overwhelmed me. | 18 | 66.7 | 22.2 | 0 | 11.1 | 0 |
| Overall, I find the program feasible. | 20 | 0 | 5.0 | 5.0 | 50.0 | 40.0 |

*Abbreviations: npMPS: non-physician medical practice staff; GP: general practitioner.*

**Supplementary Table 7** Acceptance and feasibility of the intervention among caregivers

| **Questions for informal caregivers** | *n* | Not at all  /  Never  (%) | Slightly  /  Rarely  (%) | Some-what  /  Some-times (%) | Largely  /  Rather often  (%) | Highly  /  Often (%) |
| --- | --- | --- | --- | --- | --- | --- |
| How competent was the npMPS as a counselor? | 42 | 0 | 0 | 4.8 | 9.5 | 85.7 |
| How important was the home visit compared to practice-based counseling? | 38 | 21.1 | 10.5 | 10.5 | 21.1 | 36.8 |
| How important did you find your GP's involvement in planning the counseling? | 41 | 0 | 2.4 | 12.2 | 31.7 | 53.7 |
| How helpful was the npMPS counseling for you? | 40 | 5.0 | 0 | 15.0 | 32.5 | 47.5 |
| Would you recommend this counseling to others in similar situations? | 42 | 0 | 0 | 9.5 | 33.3 | 57.1 |
| Did the counseling ever feel overwhelming to you? | 42 | 78.6 | 14.3 | 4.8 | 0 | 2.4 |

*Abbreviations: npMPS: non-physician medical practice staff; GP: general practitioner.*
